# Quorum-Sensing Regulator OpaR Directly Represses Seven Protease Genes in *Vibrio parahaemolyticus*

**DOI:** 10.3389/fmicb.2020.534692

**Published:** 2020-10-29

**Authors:** San-Chi Chang, Chia-Yin Lee

**Affiliations:** Microbiology Laboratory, Department of Agricultural Chemistry, National Taiwan University, Taipei, Taiwan

**Keywords:** quorum sensing, OpaR-binding motif, *Vibrio parahaemolyticus*, ChIP-seq, proteases regulation, EMSA, DNase I footprinting

## Abstract

Proteases play a key role in numerous bacterial physiological events. Microbial proteases are used in the pharmaceutical industry and in biomedical applications. The genus *Vibrio* comprises protease-producing bacteria. Proteases transform polypeptides into shorter chains for easier utilization. They also function as a virulence factor in pathogens. The mechanism by which protease genes are regulated in *Vibrio parahaemolyticus*, an emerging world-wide human pathogen, however, still remains unclear. Quorum sensing is the communication system of bacteria. OpaR is the master quorum-sensing regulator in *V. parahaemolyticus*. In the present study, quantitative reverse transcriptase-polymerase chain reaction and protease gene promoter-fusion reporter assays revealed that OpaR represses seven protease genes—three metalloprotease genes and four serine protease genes—which are involved in environmental survival and bacterial virulence. Furthermore, the electrophoresis mobility shift assay demonstrated that OpaR is bound directly to the promoter region of each of the seven protease genes. DNase I footprinting identified the sequence of these OpaR-binding sites. ChIP-seq analyses revealed 435 and 835 OpaR-binding sites in the late-log and stationary phases, respectively. These OpaR-binding sequences indicated a conserved OpaR-binding motif: TATTGATAAAATTATCAATA. These results advance our understanding of the protease regulation system in *V. parahaemolyticus*. This study is the first to reveal the OpaR motif within *V. parahaemolyticus in vivo*, using ChIP-seq, and to provide a database for OpaR direct regulon.

## Introduction

*Vibrio parahaemolyticus* is a gram-negative bacterium that inhabits marine and estuarine environments. Most cases of seafood-borne gastroenteritis are caused by *V. parahaemolyticus* (Su and Liu, 2007). The enteral route of *V. parahaemolyticus* is the consumption of raw or undercooked shellfish. Recent reports have suggested that this bacterium is the main agent of early mortality syndrome (Pérez-Acosta et al., 2018), also known as acute hepatopancreatic necrosis syndrome, an emerging shrimp disease that causes acute mortality in Pacific white shrimp (*Litopenaeus vannamei*) (Theethakaew et al., 2017). *V. parahaemolyticus* strains have numerous virulence factors, including thermostable direct hemolysin (TDH), TDH-related hemolysin (TRH), adhesion factors, extracellular proteases, and type III secretion system effectors (Broberg et al., 2011). Two types of flagellar systems assist *V. parahaemolyticus* with swimming and swarming (Letchumanan et al., 2014).

*Vibrio parahaemolyticus* expresses extracellular metalloproteases—PrtV, VppC, and VPM—and extracellular serine proteases—VPP1, VpS37, and PrtA (Osei-Adjei et al., 2018). PrtV is a collagenase and can be inhibited by ethylenediaminetetraacetic acid (EDTA) (Yu et al., 2000), VppC acts in the early stationary phase of growth (Miyoshi et al., 2008), VPM is a putative virulence factor (Luan et al., 2007), VVP1 causes cytotoxicity and lethality in mice (Miyoshi, 2013), VpS37 exhibits gelatinolytic activity (Salamone et al., 2015), and PrtA causes abdominal hemorrhage in mice and is toxic to various mammal cells (Lee et al., 2002).

Quorum sensing (QS) is a cell–cell communication system in which bacteria use the production and detection of extracellular chemicals, known as autoinducers, to monitor cell population density (Miller and Bassler, 2001); moreover, gene expression in a microbial community is synchronized by QS (Papenfort and Bassler, 2016). Some bacterial QS-dependent extracellular proteases were used in social cheating to inhibit microbial communities (Whiteley et al., 2017). OpaR and AphA are two QS regulators of *V. parahaemolyticus* (Gode-Potratz and McCarter, 2011; Sun et al., 2012). In low cell density, AphA controls hundreds of target genes (van Kessel et al., 2013a), whereas OpaR is a dominant regulator in high cell density (Gode-Potratz and McCarter, 2011; Burke et al., 2015). OpaR and AphA repress each other’s gene expression (Sun et al., 2012; Zhang et al., 2012).

Virulence proteases are associated with the QS system in *Vibrio* spp. Hemagglutinin/protease (HAP) is a well-known virulence factor of *V. cholerae* (Silva et al., 2003; Liang et al., 2007; Benitez and Silva, 2016) and is regulated by the QS regulator HapR (Wang et al., 2011; Dorman and Dorman, 2018). A previous study reported the purification of a novel serine protease of *V. cholerae*, which evidently plays a role in hemorrhagic response in the rabbit ileal loop model (Syngkon et al., 2010). Moreover, the serine protease VvpS (Lim et al., 2011) plays a vital role in *V. vulnificus* autolysis and is activated by the QS regulator SmcR (Kim et al., 2015). OpaR, the QS regulator of *V. parahaemolyticus*, promotes the expression of PrtA, which is an extracellular alkaline serine protease (Chang and Lee, 2018). OpaR also regulates the quantity of extracellular PrtA (Chang and Lee, 2018). In addition, QS induces the gene expression of Asp (Rui et al., 2009), which is an extracellular alkaline serine protease from *V. alginolyticus* and the cause of vibriosis in *Lutjanus erythopterus* (Cai et al., 2007).

Nutrient intake is an essential part of the bacterial life cycle—pathogens ingest nutrient molecules from host cells. A certain minimum level of protease is indispensable for the survival and growth of organisms; however, our understanding of the regulation of protease genes in *V. parahaemolyticus* is far from comprehensive. Herein, we used ChIP-seq to identify OpaR-binding sites on the genome of *V. parahaemolyticus* no. 93 (VP93), which is a *tdh*^–^ and *trh*^–^ strain. We present a conserved OpaR-binding motif and propose that OpaR directly represses seven protease genes of *V. parahaemolyticus*.

## Results

### ChIP-Seq Data Analysis Revealed a Conserved OpaR-Binding Motif

*Vibrio parahaemolyticus* can be found in multiple cell types, including free-swimming forms and colony formations attached to inert surfaces such as shellfish (Letchumanan et al., 2014). A previous study utilized whole transcriptome next-generation sequencing (RNA-Seq) for analyzing the OpaR regulon in *V. parahaemolyticus*, which was cultured on a solid growth medium. RNA-Seq data were further validated through qRT-PCR for a select subset of transcription factor genes that were highly regulated by OpaR (Burke et al., 2015). In this study, we investigated the regulation of the protease genes of *V. parahaemolyticus*, which was cultured in a liquid growth medium.

**FIGURE 1:**
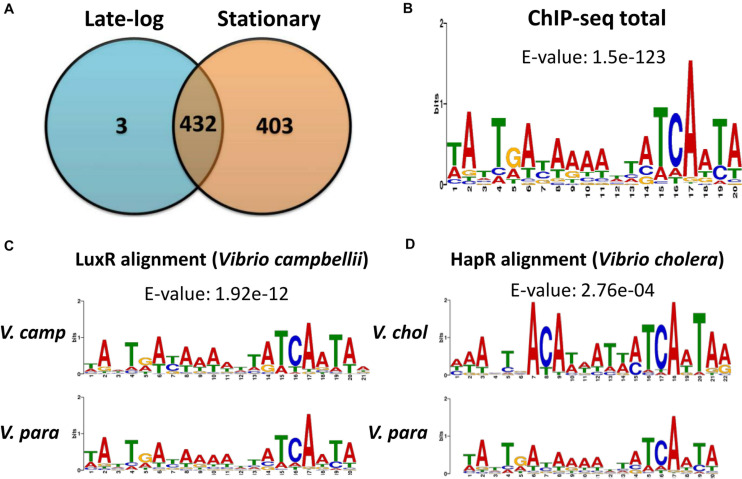
ChIP-seq data revealed *in vivo* OpaR-binding sites in *Vibrio parahaemolyticus*. **(A)** Distribution of OpaR-binding sites of ChIP-seq in the late-log and stationary phases. **(B)** Conserved OpaR-binding motif was identified from 838 OpaR-binding sequence of ChIP-seq data and analyzed using Multiple Expectation Maximization for Motif Elicitation. The height of each base represents its frequency. Conserved OpaR-binding motif aligned using TOMTOM (http://memesuite.org//tools/tomtom) with other *Vibrio* quorum-sensing regulators: **(C)** LuxR of *V. campbellii* and **(D)** HapR of *V. cholera*.

We first inserted a FLAG-tag in front of the *opaR* gene on the VP93 chromosome. Western blot results demonstrated that FLAG-OpaR is steadily expressed from the early-log to late-stationary phases (Supplementary Figure 1). We collected bacterial cells from the late-log (OD_600_ 2.8) and stationary (OD_600_ 4) phases to perform ChIP-seq assays. FLAG-OpaR was bound to the genome and cross-linked by anti-FLAG beads; the genome was sheared in 300-bp fragments, and immunoprecipitated DNA was purified and sequenced. Reads were obtained from independent ChIP-seq assays in late-log and stationary phases. The input control of ChIP-seq was the VP93 genome without anti-FLAG antibody enrichment. All reads were mapped to the VP93 genome. Our ChIP-seq data (NCBI Gene Expression Omnibus database, GEO Accession GSE122479) revealed the presence of 435 and 835 OpaR-binding sites in late-log and stationary phases, respectively; there were 432 overlapping OpaR-binding sites between the two phases (Figure 1A).

OpaR regulates nine transcription factors in *V. parahaemolyticus*: CpsR, the Crl family, the Ars family, the CsgD/VpsT family, the FhlA family, the AsnC family, ExsA, AphA, and LafK (Burke et al., 2015). Moreover, transcriptional regulation of *opaR*, *qrr2–4* (encoding QS regulatory small RNAs), and *aphA* by OpaR has been reported in *V. parahaemolyticus* (Zhang et al., 2012). In this study, we used Multiple Expectation Maximization for Motif Elicitation (MEME)^1^ to analyze 838 OpaR-binding sequences, which showed a conserved OpaR-binding motif (TATTGATAAAATTATCAATA) (Figure 1B). From genes situated near to OpaR-binding sites, we identified seven protease genes that might be regulated by OpaR in *V. parahaemolyticus*. The ChIP-seq-defined regions of the seven protease genes are listed in Table 1. In addition, we used TOMTOM^2^ to align the conserved OpaR-binding motif with other homologs of QS regulators in *Vibrio* spp., namely LuxR (Figure 1C) and HapR (Figure 1D). We observed that the OpaR-binding motif showed a high similarity with other QS regulator motifs. Comparison of the three QS regulator motifs—the conserved OpaR-binding motif, LuxR motif, and HapR motif (Figure 1)—indicated that TATTGATAAAATTATCAATA is the most conserved binding region of QS regulators in *Vibrio* spp.

**TABLE 1:** OpaR-binding positions.

Protease gene	ChIP-seq defined region^a^	EMSA probe^b^		Footprinting defined region^a^	
*lytM*	log −428 to −57	P1	−428 to −315	(1)	−427 to −408
	sta −447 to −77	P2	−237 to −152	(2)	−218 to −199
*mcp02*	log −405 to −142	P1	−405 to −207	(1)	−323 to −303
	sta −440 to −90				
*m6 protease*	log −444 to −123	P1	−444 to −296	(1)	−340 to −320
	sta −461 to −98	P2	−282 to −146	(2)	−185 to −165
*serine protease*	log −257 to +44	P1	−151 to −24	(1)	−84 to −65
	sta −270 to +36				
*degS*	log −169 to +37	P1	−164 to −18	(1)	−127 to −108
	sta −143 to +34			(2)	−113 to −94
*protease II*	sta −371 to −133	P1	−371 to −251	(1)	−285 to −266
		P2	−242 to −135	(2)	−200 to −181
*periplasmic protease*	sta +1011 to +1224	P1	+938 to +1064	(1)	+1011 to +1030

*^*a*^Nucleotide positions are numbered relative to the translational start site (+1) of every protease gene. ^*b*^EMSA, electrophoretic mobility shift assays.*

### OpaR Occupies Six Promoters and One 3′ Region of Protease Genes and Their Expression Through qRT-PCR

Table 2 reveals the functions of seven putative OpaR-regulated protease genes classified using Gene Ontology (GO) and Clusters of Orthologous Groups (COG) annotations. These seven genes could be classified into two categories: metalloendopeptidases (LytM, Mcp02, M6 protease) and serine-type endopeptidases (serine protease, DegS, periplasmic protease, protease II). Moreover, they could be classified based on their cellular locations: inner membrane protease, DegS (VP0432) (Gottesman, 2017); periplasmic protease (VP2032) (Baird et al., 1991); and extracellular proteases, LytM (VPA1649) (Gode-Potratz et al., 2011), Mcp02 (VPA0755), M6 protease (VP0907) (Rompikuntal et al., 2015), serine protease (VPA0449) (Chimalapati et al., 2018) and protease II (VPA1467). Three of these—namely Mcp02, serine protease and protease II—are secreted by the type II secretion system (Chimalapati et al., 2018).

**TABLE 2:** Functions of the seven OpaR-regulated protease genes.

Gene ID^a^ (protein)	Protein function:	
	GO category	COG category
VPA1649 (LytM) surface-induced metalloendoprotease	GO:0008233 peptidase activity	COG0739 Membrane proteins related to metalloendopeptidases
VPA0755 (Mcp02) secreted metalloprotease	GO:0046872 metal ion binding GO:0004222 metalloendopeptidase activity	COG3227 Zinc metalloprotease (elastase)
VP0907 M6 family metalloprotease domain protein	GO:0008237 metallopeptidase activity | GO:0005509 calcium ion binding	no clusters of orthologous groups (COG) classification
VPA0449 serine protease	GO:0030246 carbohydrate binding | GO:0004252 serine-type endopeptidase activity	COG1404 Subtilisin-like serine proteases
VP0432 (DegS) outer membrane stress sensor protease	GO:0004252 serine-type endopeptidase activity	COG0265 Trypsin-like serine proteases, typically periplasmic, contain C-terminal PDZ domain
VPA1467 protease II	GO:0070008 serine-type exopeptidase activity | GO:0004252 serine-type endopeptidase activity	COG1770 Protease II
VP2032 periplasmic protease	GO:0004252 serine-type endopeptidase activity	COG0616 Periplasmic serine proteases (ClpP class)

*^*a*^Gene locus number of *Vibrio parahaemolyticus* RIMD2210633 strain.*

**FIGURE 2:**
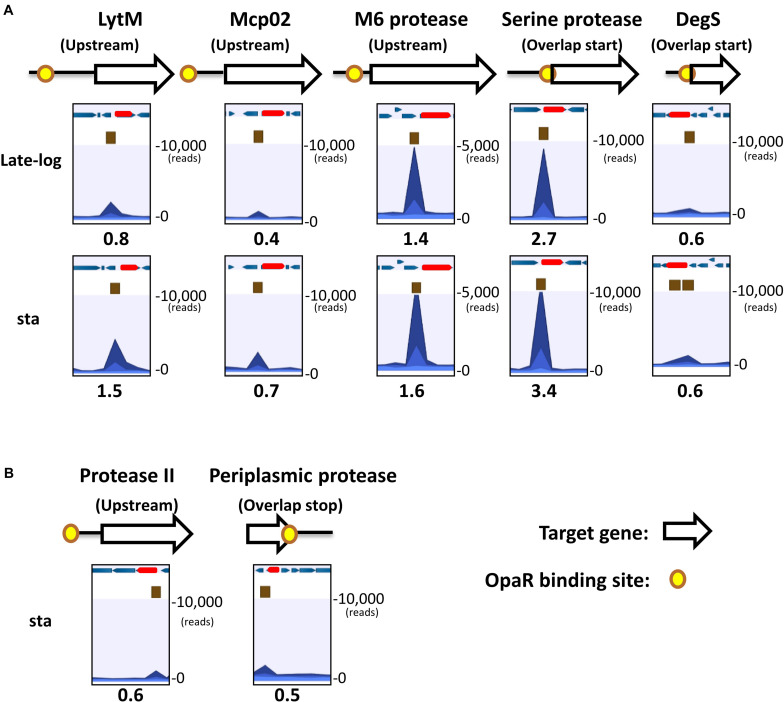
OpaR-binding peak distribution of the seven protease genes. Yellow spots represent *in vivo* OpaR-binding positions on the protease promoter or 3′region, as identified using ChIP-seq. White and red arrows denote the protease genes, and blue arrows illustrate the nearby genes. Navy blue peak represents the binding strength of the samples (ChIP-seq enriched by anti-FLAG antibody); light blue peak is the control signal (ChIP-seq without anti-FLAG antibody enrichment). Numbers below the peak diagrams represent the reads per million (RPM) value of ChIP-seq. Central OpaR-binding sites are shown in brown boxes. **(A)** OpaR bound to five protease genes—*lytM*, *mcp02*, *m6 protease*, *serine protease*, and *degS*—both in the late-log and stationary phases. **(B)** OpaR bound to both *protease II* and *periplasmic protease* only in the stationary phase.

The distribution of OpaR-binding peaks relative to the seven protease genes are shown in Figure 2. These peaks overlaid the translational start site of *serine protease* and *degS*. OpaR-binding peaks appeared on the upstream of *lytM, mcp02, M6 protease*, and *protease II*; however, one OpaR-binding peak presented at the terminator region and overlaid the translational stop site of the *periplasmic protease*. Predicted OpaR-binding sites in the promoters of *lytM, mcp02, M6 protease, serine protease*, and *degS* were found not only in the late-log phase but also in the stationary phase, whereas the OpaR occupancy in the promoter of *protease II* and terminator of *periplasmic protease* was only found in the stationary phase. Normalized reads per million (RPM) values [total reads/mapped reads (millions) x region length (bp)] represent the binding strength of OpaR-binding peaks. In addition, the binding length of OpaR ranged from 177 bp (*degS*) to 372 bp (*lytM*) (Table 1).

**FIGURE 3:**
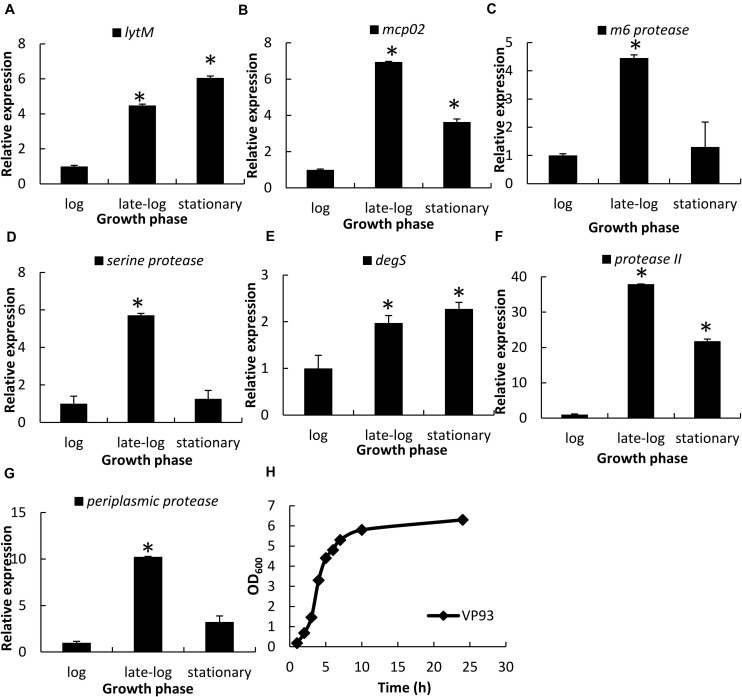
Protease gene expression levels at different growth phases, as examined using qRT-PCR. Total RNA isolated from VP93 at log (OD_600_ 1.7), late-log (OD_600_ 2.8), and stationary (OD_600_ 4) phases was purified and analyzed using specific primer sets for qRT-PCR: **(A)** *lytM*, **(B)** *mcp02*, **(C)** *m6 protease*, **(D)** *serine protease*, **(E)** *degS*, **(F)** *protease II*, and **(G)** *periplasmic protease*. **(H)** Growth curves of VP93 monitored by measuring OD_600_ at every growth stage. Values represent mean of three independent experiments, and error bars indicate standard deviation. All values were relative to the log phase, which had the value of 1. Asterisks denote statistical significance (*p < 0.01).

Each protease gene expression levels at different growth phases were examined using qRT-PCR. Genes encoding LytM (Figure 3A), Mcp02 (Figure 3B), DegS (Figure 3E), and protease II (Figure 3F) were expressed from late-log to stationary phases, whereas those encoding M6 protease (Figure 3C), serine protease (Figure 3D), and periplasmic protease (Figure 3G) were primarily expressed during the late-log phase. Growth curves of VP93 were monitored by measuring OD_600_ at every growth stage (Figure 3H). Therefore, we used late-log and stationary phase cultures to study the correlation between OpaR and the seven protease genes.

### OpaR Is a Direct Repressor of the Seven Protease Genes Validated by qRT-PCR, Promoter-Fusion *luxAB* Reporter Assays, and Electrophoretic Mobility Shift Assay

**FIGURE 4:**
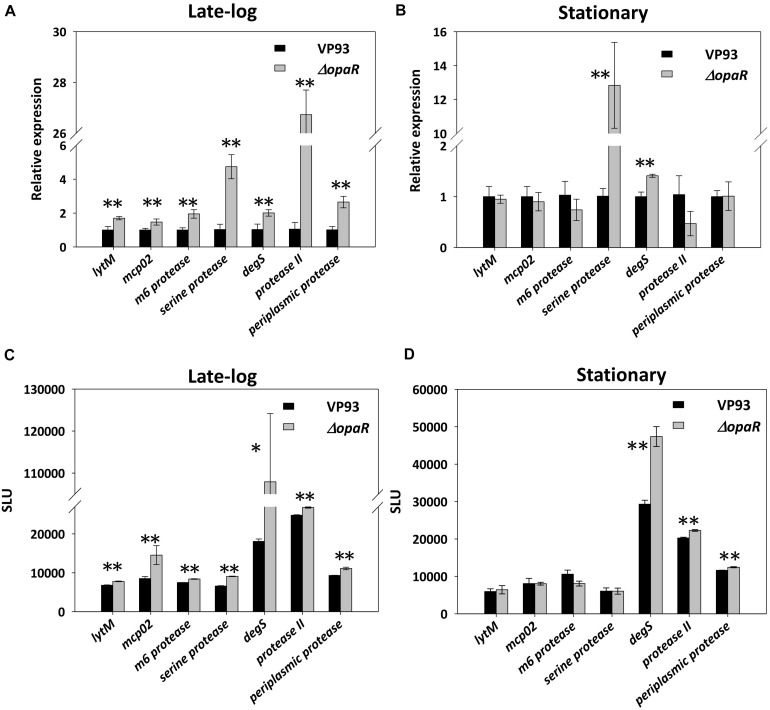
OpaR regulates the expression of the seven protease genes. Total RNA isolated from the VP93 and *ΔopaR* strains was purified, and gene expression was detected via qRT-PCR at the **(A)** late-log (OD_600_ 2.8) and **(B)** stationary (OD_600_ 4) phases. A luciferase assay was performed using VP93 and *ΔopaR* strains with the protease promoter-*luxAB* or 3’region-*luxAB* fusion plasmid at the **(C)** late-log (OD_600_ 2.8) and **(D)** stationary (OD_600_ 4) phases. Values represent mean of three independent experiments, and error bars indicate standard deviation. Asterisks denote statistical significance (*p < 0.05, **p < 0.01).

We used qRT-PCR to measure the relative transcript levels of seven protease genes in wild-type VP93 and mutant *ΔopaR* strains at late-log and stationary-phase growth. Our results revealed that OpaR represses the expression of genes encoding LytM (1.9-fold, *p* = 0.0069), Mcp02 (1.5-fold, *p* = 0.0058), M6 protease (2.0-fold, *p* = 0.0074), Serine protease (4.8-fold, *p* = 0.0054), DegS (2.1-fold, *p* = 0.0085), Protease II (25.7-fold, *p* = 0.0001), and Periplasmic protease (2.6-fold, *p* = 0.0072) in the late-log phase (Figure 4A). Moreover, this differential gene expression was also observed in the stationary phase for both Serine protease (repressed 12.7-fold, *p* = 0.0060) and DegS (repressed 1.5-fold, *p* = 0.0018) (Figure 4B).

To determine the role of these OpaR-occupied positions of protease genes from Figure 2, the promoter-fusion *luxAB* reporter assay was conducted. We amplified the aforementioned putative OpaR-binding sequences of each protease gene and cloned them into the promoter-less vector pSAluxAB. Bioluminescence was measured for the wild-type VP93 and the mutant *ΔopaR* strains at late-log and stationary phases. The results revealed that OpaR represses the bioluminescence expression for protease genes through OpaR-binding sites in the late-log phase, including *lytM* (1.2-fold, *p* = 0.0002), *mcp02* (1.8-fold, *p* = 0.0089), *m6 protease* (1.1-fold, *p* = 0.0017), *serine protease* (1.4-fold, *p* = 0.00001), *degS* (6.0-fold, *p* = 0.0106), *protease II* (1.1-fold, *p* = 0.0001), and *periplasmic protease* (1.2-fold, *p* = 0.0094) (Figure 4C). However, the expressions of *degS* (1.6-fold, *p* = 0.0030), *protease II* (1.1-fold, *p* = 0.0003), and *periplasmic protease* (1.1-fold, *p* = 0.0076) were regulated by OpaR in the stationary phase (Figure 4D).

**FIGURE 5:**
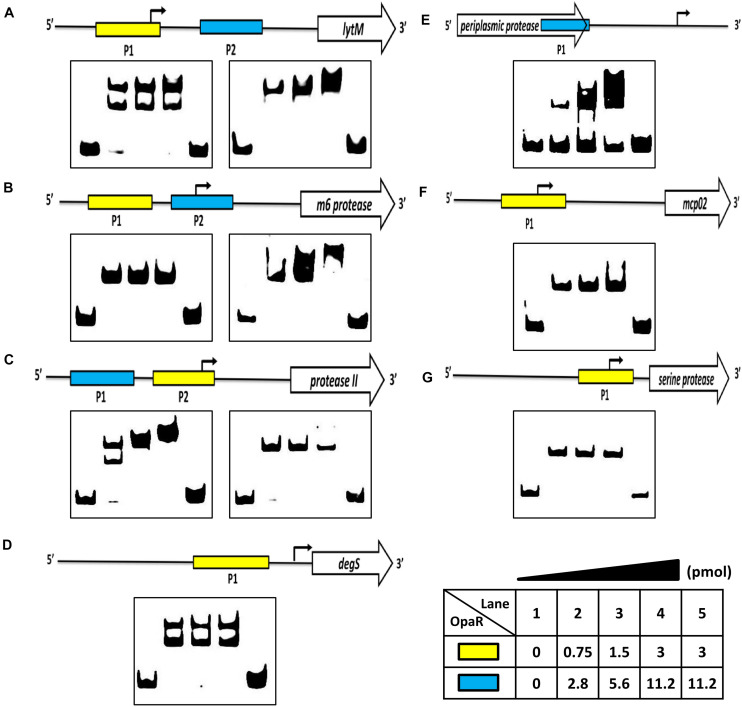
OpaR bound to the promoter region of the protease genes *in vitro*. An OpaR-binding reaction was performed with the promoter sequences of the protease genes. **(A)** *lytM*, **(B)** *m6 protease*, **(C)** *protease II*, **(D)** *degS*, **(E)** *periplasmic protease*, **(F)** *mcp02*, and **(G)** *serine protease*. The more sensitive electrophoretic mobility shift assay (EMSA) probes of each protease gene are labeled with a yellow box. The blue box indicates the other EMSA probe. Digoxigenin-labeled EMSA probes were incubated without (lane 1) or with increasing concentrations of purified OpaR (lanes 2–5) for the yellow and blue boxes. Lane 5 included 100 times more unlabeled probes as competitors.

The electrophoretic mobility shift assay (EMSA) was performed to evaluate the potential of the direct binding of OpaR to the promoters of the protease genes. We amplified the DNA fragments as the probes (P1 and P2) for each target gene, and the positions of EMSA probes are listed in Table 1. EMSA demonstrated that OpaR bound six target protease gene promoters (Figure 5). Two OpaR-binding sites (P1 and P2) were present within *lytM, m6 protease*, and *protease II* promoter regions (Figures 5A–C), and only one (P1) was present on *degS, mcp02*, and *serine protease* promoter regions (Figures 5D,F,G). Indeed, in OpaR EMSA, two shifted bands were observed in the P1 region of *lytM, protease II*, and *degS* promoters (Figures 5A,C,D). These bands are attributable to the presence of more than one individual binding sites in the promoter P1 region; they may also represent two or more different states of DNA binding. Notably, we also observed a *periplasmic protease* 3’ P1 region that shifted in the presence of 2.8–11.2 pmol of OpaR (Figure 5E). OpaR did not bind to 16S rDNA as the negative control of EMSA (data not shown).

### Identification of OpaR-Binding Sequences in the Seven Protease Genes

**FIGURE 6:**
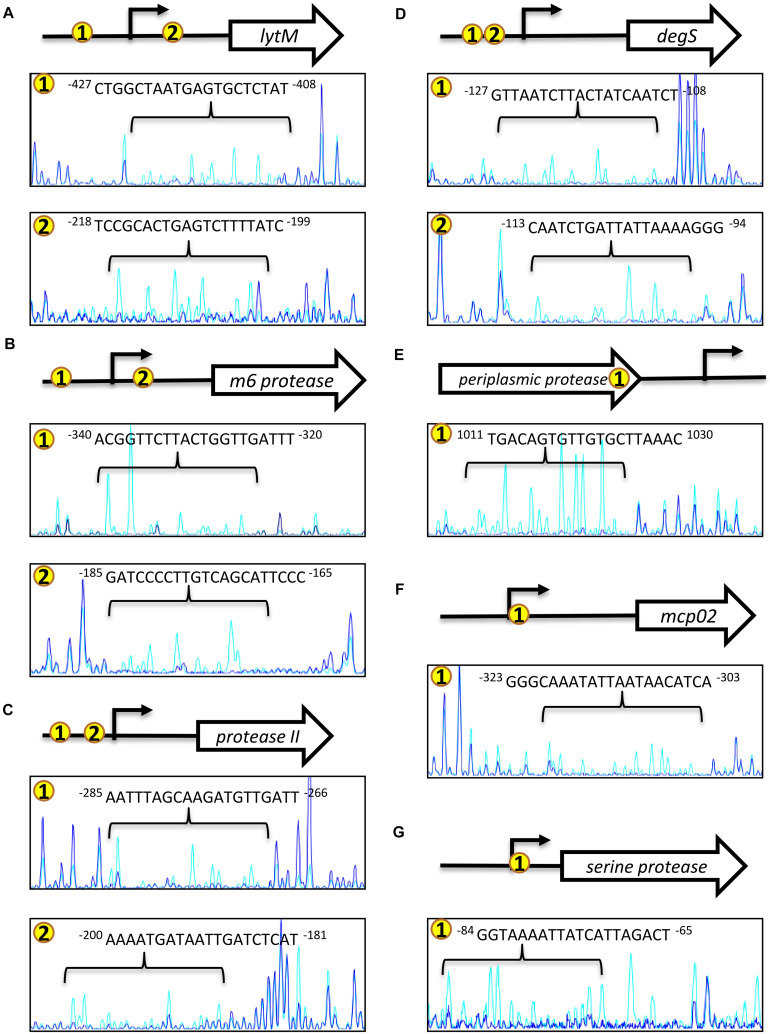
OpaR-binding sequences determined using footprinting assays. The footprinting assay involved capillary electrophoresis analyses on each protease gene promoter sequence containing the ChIP-seq and EMSA-defined OpaR-binding region. Capillary electrophoresis diagrams are shown: **(A)** *lytM*, **(B)** *m6 protease*, **(C)** *protease II*, **(D)** *degS*, **(E)** *periplasmic protease*, **(F)** *mcp02*, and **(G)** *serine protease*. Number in yellow circles on the gene promoter and 3’region are consistent with those in the peak diagram. The blue peak shows footprinting results with OpaR. The light blue peak is the control (without OpaR). The arrow (→) represents the predicted transcriptional start site of the protease genes.

We amplified the promoter region containing all the EMSA probe sequences of each protease gene to design footprinting probes. The 5′-end of the sense and antisense footprinting probes were labeled with 6-carboxyfluorescein. Labeled probes were incubated with purified His-OpaR and digested by DNase I. Capillary electrophoresis was used to analyze the digested DNA fragments. The OpaR-binding sequences on the seven protease genes are listed in Table 3, and the specific OpaR-binding positions are presented in Figure 6. The OpaR-binding position was defined by the difference of peak observed between the control without OpaR and samples with OpaR. We found two OpaR-binding sequences for *lytM*, *m6 protease*, *protease II*, *degS* (Figures 6A–D). Capillary electrophoresis analysis identified one OpaR-binding sequence for *periplasmic protease*, *mcp02* and *serine protease* (Figures 6E–G). Notably, upon aligning the OpaR-binding sequences obtained from the DNase I footprinting assay with the EMSA probe sequences, *degS* EMSA revealed a double band shift, and we could locate and identify two OpaR-binding sequences in the DNase I footprinting assay result (Figures 5D, 6D).

**TABLE 3:** OpaR-binding sequences on protease gene promoter regions as revealed using DNase I footprinting assay.

Protease gene	Sense sequence	Antisense sequence
***lytM***		
(1)	CTGGCTAATGAGTGCTCTAT	ATAGAGCACTCATTAGCCAG
(2)	TCCGCACTGAGTCTTTTATC	GATAAAAGACTCAGTGCGGA
***mcp02***		
(1)	GGGCAAATATTAATAACATCA	TGATGTTATTAATATTTGCCC
***m6 protease***		
(1)	ACGGTTCTTACTGGTTGATTT	AAATCAACCAGTAAGAACCGT
(2)	GATCCCCTTGTCAGCATTCCC	GGGAATGCTGACAAGGGGATC
***serine protease***		
(1)	GGTAAAATTATCATTAGACT	AGTCTAATGATAATTTTACC
***degS***		
(1)	GTTAATCTTACTATCAATCT	AGATTGATAGTAAGATTAAC
(2)	CAATCTGATTATTAAAAGGG	CCCTTTTAATAATCAGATTG
***protease II***		
(1)	AATTTAGCAAGATGTTGATT	AATCAACATCTTGCTAAATT
(2)	AAAATGATAATTGATCTCAT	ATGAGATCAATTATCATTTT
***periplasmic protease***		
(1)	TGACAGTGTTGTGCTTAAAC	GTTTAAGCACAACACTGTCA

To further verify whether the OpaR-binding site appeared at the *periplasmic protease* 3′region, we performed sequence analysis using ARNold program (Naville et al., 2011). The result revealed that a Rho-independent transcriptional terminator (intrinsic terminator) was present and located 29 to 50-nt downstream of the *periplasmic protease* stop codon. DNase I footprinting assay result showed that one OpaR-binding sequence located 33 to 52-nt upstream of the stop codon (Figure 6E). OpaR binding close to the stop coden of the ORF may block transcriptional elongation and result in the target gene repression. These results conclude that OpaR binds to the ORF near 3′ region to repress *periplasmic protease* expression.

### Diagram of QS-Dependent Protease Regulation

**FIGURE 7:**
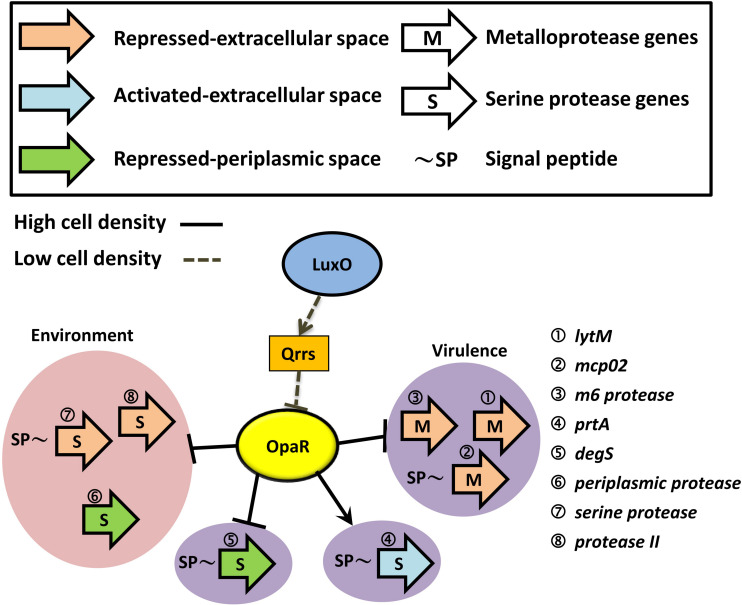
Schematic of how OpaR directly regulates protease genes. Black arrows represent positive regulation of protease genes by OpaR. Protease genes were categorized depending on activation/repression, periplasmic/extracellular localization, and metalloprotease/serine protease. Proteases that might involve bacterial virulence are depicted on a purple background. The pink region includes the environmental survival genes. Dashed and solid lines indicate the regulation pathway at a low and high cell density, respectively. Signal peptides were predicted by SignalP v5.0 (Armenteros et al., 2019) and are labeled as ∼SP.

At a high cell density, OpaR repressed three groups of proteases: extracellular metalloprotease, extracellular serine protease, and periplasmic serine protease. However, PrtA (VPA0227) is an extracellular serine protease that was promoted by OpaR, as reported in a previous study (Chang and Lee, 2018). As shown in Figure 7, OpaR directly regulated the gene expression of the eight protease genes: three environmental survival genes—*periplasmic protease* (Baird et al., 1991), *serine protease*, and *protease II* (Kanatani et al., 1991, 1992)—and five bacterial virulence genes—*lytM* (Gode-Potratz et al., 2010), *mcp02* (Gao et al., 2010; Miyoshi, 2013), *m6 protease* (Vaitkevicius et al., 2008; Rawlings et al., 2018), *prtA* (Lee et al., 2002), and *degS* (Mathur et al., 2007).

## Discussion

A report using *in vitro* EMSA with purified His-tagged OpaR demonstrated nine direct OpaR targets (Burke et al., 2015) and indicated that MQSR matrix is too strict to predict OpaR-binding sites (Burke et al., 2015). OpaR-binding sequences have been reported among *opaR*, *qrr2*, *qrr3*, *qrr4*, *aphA*, T6SS2 genes, T3SS-associated genes, and flagellar genes (Zhang et al., 2012; Wang et al., 2013; Burke et al., 2015; Lu et al., 2019). ChIP-seq is a powerful method for investigating the binding targets of bacterial transcription factors. Our findings also revealed that 432 OpaR-binding sites overlapped between the late-log and stationary phases and that 403 OpaR-binding sites were present only in the stationary phase (Figure 1A). This finding implied that OpaR occupies more regions of the VP93 genome in the stationary phase than in the late-log phase. OpaR regulates many genes at high cell density, such as lateral flagellar genes, biofilm genes, and T3SS operon genes (Ball et al., 2017). Moreover, the global stress regulator RpoS in the stationary phase induced the expression of a QS regulator (Weber et al., 2008). Stationary-phase regulatory proteins were controlled by the QS system (Lazazzera, 2000). In the present study, we identified 838 *in vivo* OpaR-binding sites using ChIP-seq assay and suggested that the most conserved OpaR-binding motif was TATTGATAAAATTATCAATA (Figure 1).

The seven protease genes mentioned in this study have a unique function in *V. parahaemolyticus.* A previous microarray-based study in which *V. parahaemolyticus* was cultured on a solid growth medium reported that OpaR represses surface-sensing regulon and different secretion systems (Gode-Potratz and McCarter, 2011). LytM reportedly belongs to the surface-sensing regulon and is regulated by the master regulator of the lateral flagellar system LafK (Gode-Potratz et al., 2011). Mcp02 is a putative vibriolysin and causes instant cytotoxicity during infections (Gao et al., 2010). M6 protease has an M6 protease domain in the N-terminal and plays a role in bacterial environmental persistence and survival (Rawlings et al., 2018). M6 protease purified from *V. cholera* has been reported to cause cytotoxicity and degrade host tissue components (Vaitkevicius et al., 2008). DegS is involved in the periplasmic stress response against antimicrobial peptides (Mathur et al., 2007). Protease II is involved in amino acid transport and metabolism in *V. parahaemolyticus* (Chimalapati et al., 2018).

Although most binding regulations occur outside of upstream intergenic regions, target gene transcription is more often repressed by binding downstream of the proximal promoter (Babu and Teichmann, 2003). In the present study, ChIP-seq data and EMSA results revealed that OpaR binds to the 3′ end region of the periplasmic protease gene and represses the gene expression. Furthermore, from a report of OpaR RNA-seq data (Burke et al., 2015), we found some genes down regulated by OpaR at greater than 4-fold and noted that OpaR is located at the 3′end region of putatively regulated target genes from our ChIPseq data (data not shown). However, we demonstrated that opaR directly regulates the transcription of the other six protease genes through the OpaR-binding sites in their promoters.

OpaR is a homolog of LuxR, which is a QS regulator in *V. harveyi* (Pompeani et al., 2008). At a high cell density, QS regulators exhibit maximal gene expression (Waters and Bassler, 2006; Kleinman et al., 2017). They regulate the downstream genes through their activation or repression (van Kessel et al., 2013b). A recent study found that LuxR directly interacts with RNA polymerase to activate transcription of the *luxCDABE* bioluminescence genes; moreover, LuxR DNA-binding sites that are present in close proximity to the -35 region of the promoter are required for activation at some promoters (Ball and van Kessel, 2019). Notably, our recent report indicated that PrtA, which belongs to the surface-sensing regulon (Ferreira et al., 2012), is a unique OpaR-activated protease gene. The OpaR-binding sequence overlapped the -35 region of the *prtA* promoter (Chang and Lee, 2018). PrtA is also activated by LafK, which is involved in cytotoxicity and pathogenicity in *V. parahaemolyticus*.

Herein, we used a strain of *V. parahaemolyticus* that was a chromosomal FLAG-tag insertion strain, not an overexpression strain with plasmids. Therefore, the *in vivo* data of this study were affected neither by OpaR overexpression nor by plasmids exhibiting antibiotic resistance. To the best of our knowledge, this is the first study to report a conserved OpaR-binding motif, TATTGATAAAATTATCAATA, and seven OpaR-downregulated protease genes in *V. parahaemolyticus*.

## Materials and Methods

### Bacterial Strains and Growth Conditions

All bacterial strains, plasmids, and primers are listed in Supplementary Tables 1, 2. *V. parahaemolyticus* strains and their derivatives were cultured in tryptic soy bean broth containing 3% NaCl (TSB3) (Difco) at 35°C. *Escherichia coli* strains were grown in Luria–Bertani (Difco) medium at 35°C. XL1-Blue (Bullock et al., 1987) and S17-1 λ*pir* (Delorenzo et al., 1993) were used for cloning and conjugation, respectively. Mutant and reporter strains were derived from VP93. When necessary, antibiotics were added to the culture medium: ampicillin, 100 μg ml^–1^; kanamycin, 25 μg ml^–1^; and chloramphenicol, 20 μg ml^–1^ for *E. coli* and 5 μg ml^–1^ for *V. parahaemolyticus*.

### FLAG-Tag Insertion Before the OpaR Gene

We constructed a FLAG-tag insertion strain using homologous recombination. First, a FLAG-tag (DYKDDDDK) was inserted after the translational start site of *opaR* using overlapping PCR. The FLAG-*opaR* fragment was then ligated to pDS132, a suicide vector (Philippe et al., 2004), to yield the pDS132–FLAG-*opaR* plasmid. This mobilizable plasmid was transferred from *E. coli* S17-1 λ*pir* to VP93 via conjugation (Schafer et al., 1990). The conjugants were selected from a TSB3 Cm plate for the first step of the homologous recombination. Next, thiosulfate–citrate–bile salts–sucrose plates containing 6% sucrose were used to select the second homologous recombination conjugants. PCR amplification and sequencing were used to confirm the generation of the FLAG-tag insertion strain.

### Western Blotting

FLAG-OpaR/VP93 strain culture was collected at every growth stage by centrifugation. Total proteins of the FLAG-OpaR/VP93 strain were separated using 12.5% SDS-PAGE gels and transferred to PVDF membranes (Merck Millipore, Germany). The membrane was blotted with anti-FLAG antibody as the primary antibody. The secondary antibody is an anti-rabbit IgG HRP-linked antibody. Immobilon Western Chemiluminescent Horseradish Peroxidase Substrate (Merck Millipore, Germany) was added, and signals were detected using a chemiluminescence image system, MutiGel-21 (Bio Pioneer Tech, Taipei, Taiwan).

### ChIP-Seq and Analyses

FLAG-OpaR/VP93 was grown to the late-log (OD_600_ 3) and stationary (OD_600_ 4.2) phases. Next, the bacterial culture was incubated with 1% formaldehyde for 20 min at 25°C, and cross-linking was ceased using 0.5 M glycine. Bacterial cells were then harvested by centrifugation, washed three times with Tris-buffered saline buffer, and resuspended in a lysis buffer [10 mM Tris, 100 mM NaCl, 1 mM EDTA, 0.5 mM EGTA, 0.1% deoxycholate, and 0.5% N-lauroylsarcosine (pH 8.0)] containing a protease inhibitor cocktail (Sigma). The lysed cells were sonicated using Bioruptor Pico^®^ (Diagenode) for 20 cycles. Cell debris was removed by centrifugation; supernatant DNA fragments ranged from 300 bp to 500 bp. Anti-FLAG antibody beads (Sigma) were incubated with supernatant DNA fragments overnight at 4°C. After the ChIP reaction, samples were washed five times with a radioimmunoprecipitation assay buffer [50 mM hydroxyethyl piperazineethanesulfonic acid buffer, 500 mM LiCl, 1 mM EDTA, 1% Non-idet P-40, and 0.7% deoxycholate (pH 7.5)] and twice with Tris-buffered saline buffer. ChIP DNA was eluted in an elution buffer (50 mM Tris-EDTA buffer, 1% EDTA, pH 7.5) at 65°C for 30 min to de-cross-link. Samples were treated with RNase A and proteinase K (Dong and Mekalanos, 2012), and ChIP DNA was then purified using the Agencourt AMPure XP PCR Purification Kit (Beckman Coulter). Libraries for ChIP-seq were prepared using the Ovation Ultralow Library System V2 1–96 (NuGEN). Sequencing was performed using the HiSeq 2000 sequencing system (Illumina). ChIP-quantitative PCR was performed using SYBR green (BioRad) to determine the quality (S/N ratio) of ChIP-DNA. AphA and VPA0606 were used as positive and negative controls, respectively (Burke et al., 2015).

We used ”Map Reads to Reference” in CLC Genomics Workbench v9.5 platform conducting Reference mapping. We also used ”Transcription factor ChIP-Seq Analysis” conducting the peak finding. Maximum P-value for peak calling is < 0.05. The OpaR-binding motif was generated using MEME^1^ (Bailey et al., 2009). TOMTOM, a web-based tool^2^ (Bailey, 2011), was used to align the conserved OpaR-binding motif with other homologs. The ChIP-seq data could be obtained with the GEO accession number GSE122479.

### His_6_-OpaR Protein Expression and Purification

His_6_-OpaR protein was expressed and purified as previously described (Chang and Lee, 2018). Briefly, to generate the pET28a–*opaR* plasmid, *opaR* was cloned into the six-His-tag expression vector pET28a (Novagen) (Supplementary Table 1). The clone was transformed into *E. coli* BL21 (DE3) to express OpaR with an N-terminal fusion tag. *E. coli* BL21 (DE3) with pET28a–*opaR* was grown in 200 mL Luria–Bertani broth supplemented with 25 μg mL^–1^ kanamycin at 37°C. When OD_600_ reached 0.4–0.6, 1 mM isopropyl β-D-1-thiogalactopyranoside was used to induce His_6_-OpaR protein expression at 30°C for 4 h. The cells were then collected and disrupted using the Constant Cell Disrupter System (Constant Systems Ltd., Daventry, United Kingdom), and nickel–nitrilotriacetic acid chromatography (GE Healthcare, Chicago, IL, United States) was used to purify His_6_-OpaR. His_6_-OpaR purity was confirmed using sodium dodecyl sulfate polyacrylamide gel electrophoresis. Proteins were quantified using the method described by Bradford (Bradford, 1976).

### Quantitative Reverse-Transcriptase PCR

We collected the bacterial cultures from different growth phases of VP93 and *ΔopaR*. Cells were mixed with RNAprotect Bacteria Reagent (Qiagen) and centrifuged at 5000 *g* at 4°C for 15 min. Total RNA was isolated using TRIzol. Reverse transcription–PCR (RT-PCR) was performed using HiScript I Reverse Transcriptase (Bionovas Biotechnology), according to the manufacturer’s instructions, and real-time quantitative reverse-transcriptase (qRT-PCR) was performed using SYBR Green Master Mix (iQTM SYBR Green^®^ Supermix, BioRad) on a BioRad CFX96 system. Relative expression values were determined using 2^–(Δ^ ^*Ct* Target^^–^^Δ^ ^*Ct* Reference)^. Ct is the fractional threshold cycle, and the reference was 16S ribosomal RNA gene. The specific primer sets used for qRT-PCR are listed in Supplementary Table 2.

### Promoter-*luxAB* Fusion Plasmid and Luciferase Assay

The promoter regions of the six OpaR-regulated protease genes and one 3’ end region of *periplasmic protease* were amplified using specific primer sets (Supplementary Table 2). The promoter fragments were cloned into a promoter-less vector pSAluxAB (Supplementray Table 1), with *Sma*I and *Xba*I restriction sites. These plasmids were transformed into VP93 and Δ*opaR* by using the Gene Pulser Xcell Electroporation System (BioRad). Luciferase activity was measured by adding n-decanol [final concentration 0.001% (vol/vol)] as the substrate to 2 mL bacterial cultures. Luminescence was detected on a Spectrofluorometry F-2500 device (Hitachi, Tokyo, Japan) and is reported as specific light units (SLU; relative luminescent light units per second per milliliter per OD_600_ unit).

### EMSA

The specific primer sets used to amplify EMSA probes are listed in Supplementary Table 2. These probes were labeled with digoxigenin-11-ddUTP at their 3′-ends using the second generation DIG Gel Shift Kit (Roche Applied Sciences, Mannheim, Germany). In the binding reaction, 0.4 ng labeled fragment was incubated with various quantities of purified His-OpaR protein in 20 μL binding buffer [100 mM hydroxyethyl piperazineethanesulfonic acid (pH 7.6), 5 mM EDTA, 50 mM (NH_4_)_2_SO_4_, 5 mM dithiothreitol, 1% (wt/vol) Tween 20, and 150 mM KCl] at 25°C for 30 min. In competition analyses, the protein–DNA complex was mixed with 100 ng unlabeled fragment. These samples were separated using 6% native polyacrylamide gel electrophoresis in 0.5 × Tris-borate-EDTA buffer and transferred by electroblotting to a positively charged nylon membrane. Finally, immunological treatment and chemiluminescent signal detection were performed according to the instructions for the second generation DIG Gel Shift Kit (Roche Applied Sciences, Mannheim, Germany).

### DNase I Footprinting Assay

The DNase I footprinting assay was performed using the method of Zianni et al. (2006). The probes of the DNase I footprinting assay were amplified using specific primer sets (Supplementary Table 2). These probes contained the promoter regions or the 3′ end region of the protease genes. The 5′-ends of the sense and antisense probes were labeled with 6-carboxyfluorescein. In the binding reaction, 75 ng labeled probe and various quantities of His-OpaR protein were mixed in 20 μL binding buffer (second generation DIG Gel Shift Kit) and incubated at 25°C for 30 min. We then added 0.015 U DNase I (Thermo scientific, Germany) at 25°C for 1 min to digest the DNA–protein complex and ceased this reaction using 2.77 μL stop solution (5 mM EDTA) at 65°C for 10 min. The Agencourt AMPure XP PCR Purification Kit (Beckman Coulter) was used to purify the digested DNA fragments. Subsequently, 1 μL digested DNA fragments were added to a mixture of 8.5 μL highly deionized formamide and 0.5 μL GeneScan^TM^ LIZ 600 Size Standard (Applied Biosystems). The samples were analyzed using the ABI 3730xl DNA Analyzer (Applied Biosystems), and the results were analyzed using Peak Scanner Software v1.0 (Applied Biosystems).

### Statistical Analysis

Statistical differences between the three samples were measured using Student’s *t*-test with a two-tailed distribution. *P* < 0.05 or *P* < 0.01 indicated statistical significance.

## Data Availability Statement

The ChIP-seq data have been submitted to the National Center for Biotechnology Information’s Gene Expression Omnibus (GEO) database (accession number: GSE122479).

## Author Contributions

S-CC and C-YL designed the study. S-CC performed the experiments and data analyses. C-YL supervised and coordinated the project. S-CC wrote the preliminary draft of this manuscript and C-YL revised the manuscript. Both authors have reviewed and approved the manuscript.

## Conflict of Interest

The authors declare that the research was conducted in the absence of any commercial or financial relationships that could be construed as a potential conflict of interest.

## Footnotes

1 http://meme-suite.org/

2 http://meme-suite.org//tools/tomtom
