# Supplementary material for: Quorum-Sensing Regulator OpaR Directly Represses Seven Protease Genes in Vibrio parahaemolyticus
Source: Front Microbiol. 2020 Oct 29;11:534692. doi: 10.3389/fmicb.2020.534692 (PMC7658014; doi:10.3389/fmicb.2020.534692)
Supplement: Supplementary file 1 [file Data_Sheet_1.pdf]

Quorum sensing regulator OpaR directly represses seven protease genes in *Vibrio parahaemolyticus*

Supplementary materials:

Table S1. Bacterial strains and plasmids used in this study.

Table S2. Primers used in this study.

Figure S1. Expression levels of FLAG-OpaR at different growth phases.

Table S1 Bacterial strains and plasmids used in this study

**Strain or plasmid**	**Description**[a]	**Reference**
**Strain**		
V. parahaemolyticus		
no. 93	*tdh*⁻ *trh*⁻	(Yu & Lee, 1999)
ΔopaR	no.93*ΔopaR*	(Chang & Lee, 2018)
*FLAG-opaR/*VP93	no.93 *FLAG-opaR*	This study
pSAluxAB/VP93	no.93 with pSAluxAB	This study
pSAluxAB/*ΔopaR*	*ΔopaR* with pSAluxAB	This study
pSAluxAB-*lytM* promoter /VP93	no.93 with pSAluxAB-*lytM* promoter	This study
pSAluxAB-*lytM* promoter /*ΔopaR*	*ΔopaR* with pSAluxAB-*lytM* promoter	This study
pSAluxAB-*mcp02* promoter /VP93	no.93 with pSAluxAB-*mcp02* promoter	This study
pSAluxAB-*mcp02* promoter /*ΔopaR*	*ΔopaR* with pSAluxAB-*mcp02* promoter	This study
pSAluxAB-*m6 protease* promoter /VP93	no.93 with pSAluxAB-*m6 protease* promoter	This study
pSAluxAB-*m6 protease* promoter /*ΔopaR*	*ΔopaR* with pSAluxAB-*m6 protease* promoter	This study
pSAluxAB-*serine protease* promoter /VP93	no.93 with pSAluxAB-*serine protease* promoter	This study
pSAluxAB-*serine protease* promoter /*ΔopaR*	*ΔopaR* with pSAluxAB-*serine protease* promoter	This study
pSAluxAB-*degS* promoter /VP93	no.93 with pSAluxAB-*degS* promoter	This study
pSAluxAB-*degS* promoter /*ΔopaR*	*ΔopaR* with pSAluxAB-*degS* promoter	This study
pSAluxAB-*protease II* promoter /VP93	no.93 with pSAluxAB-*protease II* promoter	This study
pSAluxAB-*protease II* promoter /*ΔopaR*	*ΔopaR* with pSAluxAB-*protease II* promoter	This study
pSAluxAB-*periplasmic protease* 3'region /VP93	no.93 with pSAluxAB-*periplasmic protease* 3'region	This study
pSAluxAB-*periplasmic protease* 3'region /*ΔopaR*	*ΔopaR* with pSAluxAB-*periplasmic protease* 3'region	This study
Escherichia coli		
XL1-Blue	*rec*A1*end*A1 *gyr*A96 *thi*$^{-1}$ *hsd*R17 *sup*E44 *rel*A1 *lac* [F' *pro*AB *lac*I^{q} *Δ*(*lac*Z) M15 Tn10 (*Tet*^{r})], cloning host	Strategene
S17-1 λ*pir*	*recA*::RP4-2-Tc::Mu λ *pir* R6K	(Delorenzo *et al.*, 1993)
BL21 (DE3)	F⁻ *ompT gal dcm lon hsdS*$_{B}$ (r$_{B}^{-}$ m$_{B}^{-}$) λ (DE3) [*lacI lacU*V5-T7 gene 1 *ind1 sam7 nin5*])	Novagen
XL1-Blue/pDS132-FLAG-*opaR*	XL1-Blue with pDS132-FLAG-*opaR* plasmid	This study
S17-1 λ*pir*/pDS132-FLAG-*opaR*	S17-1 λ*pir* with pDS132-FLAG-*opaR* plasmid	This study
pSAluxAB-*lytM* promoter/ XL1-Blue	XL1-Blue with pSAluxABl-*lytM* promoter	This study
pSAluxAB-*mcp02* promoter / XL1-Blue	XL1-Blue with pSAluxAB-*mcp02* promoter	This study
pSAluxAB-*m6 protease* promoter / XL1-Blue	XL1-Blue with pSAluxAB-*m6 protease* promoter	This study
pSAluxAB-*serine protease* promoter / XL1-Blue	XL1-Blue with pSAluxAB-*serine protease* promoter	This study

pSAluxAB-*degS* promoter / XL1-Blue	XL1-Blue with pSAluxAB-*degS* promoter	This study
pSAluxAB-*protease II* promoter / XL1-Blue	XL1-Blue with pSAluxAB-*protease II* promoter	This study
pSAluxAB-*periplasmic protease* 3'region/ XL1-Blue	XL1-Blue with pSAluxAB-*periplasmic protease* 3'region	This study
pET28a-*opaR/* XL1-Blue	XL1-Blue with pET28a-*opaR*	This study
pET28a-*opaR/* BL21 (DE3)	BL21 (DE3) with pET28a-*opaR*	This study
**Plasmids**		
RBC TA Vector	cloning Vector, Amp^r	RBC
pDS132	R6K ori, mobRP4, *cat, sacB* suicide vector for gene replacement, Cm^r	(Philippe *et al.*, 2004)
pET28a	expression vector, Km^r	Novagen
pSA19CP-MCS	*Vibrio parahaemolyticus* plasmid pSA19 holding MCS derived from pUC119, Cm^r	(Nomura *et al.*, 2000)
pSAluxAB	pSA19CP vector with luxAB reporter gene, Cm^r	This study
pDS132-*mopaR*	derivative of pDS132 for generating the *opaR* deletion mutant, Cm^r	This study
pDS132-FLAG-*opaR*	derivative of pDS132 for generating the FLAG-*opaR*, Cm	This study
pSAluxAB-*lytM* promoter	pSAluxAB with 740 bp *lytM* promoter for luciferase assay, Cm^r	This study
pSAluxAB-*mcp02* promoter	pSAluxAB with 622 bp *mcp02* promoter for luciferase assay, Cm^r	This study
pSAluxAB-*m6 protease* promoter	pSAluxAB with 708 bp *m6 protease* promoter for luciferase assay, Cm^r	This study
pSAluxAB-*serine protease* promoter	pSAluxAB with 679 bp *serine protease* promoter for luciferase assay, Cm^r	This study
pSAluxAB-*degS* promoter	pSAluxAB with 552 bp *degS* promoter for luciferase assay, Cm^r	This study
pSAluxAB-*protease II* promoter	pSAluxAB with 607 bp *protease II* promoter for luciferase assay, Cm^r	This study
pSAluxAB-*periplasmic protease* 3'region	pSAluxAB with 509 bp *periplasmic protease* 3'region for luciferase assay, Cm^r	This study
pET28a-*opaR*	pET28a containing *opaR* coding region, Km^r	This study

[a] Amp^r, ampicillin resistance; Cm^r, chloramphenicol resistance; Km^r, kanamycin resistance

References

Chang, S.C., and Lee, C.Y. (2018) OpaR and RpoS are positive regulators of a virulence factor PrtA in *Vibrio parahaemolyticus*. *Microbiol-Sgm* 164, 221-231.

Delorenzo, V., Eltis, L., Kessler, B., and Timmis, K.N. (1993) Analysis of *Pseudomonas* gene-products using *lacI*^{q}/*Ptrp-lac* plasmids and transposons that confer conditional phenotypes. *Gene* 123, 17-24.

Nomura, T., Hamashima, H., and Okamoto, K. (2000) Carboxy terminal region of haemolysin of *Aeromonas sobria* triggers dimerization. *Microb. Pathogenesis* 28, 25-36.

Philippe, N., Alcaraz, J.P., Coursange, E., Geiselmann, J., and Schneider, D. (2004) Improvement of pCVD442, a suicide plasmid for gene allele exchange in bacteria. *Plasmid* 51, 246-255.

Yu, M.S., and Lee, C.Y. (1999) Expression and characterization of the *prtV* gene encoding a collagenase from *Vibrio parahaemolyticus* in *Escherichia coli*. *Microbiol-Sgm* 145, 143-150.

Table S2 Primers used in this study

Primer name	Sequence (5' → 3')[a]	Source
Strain construction		
Vp2516D-A2	TAGCATGCTACCTGTATCGA	This study
Vp2516D-B1	GAGCTCTTTCAACTGTTGTTGCTTACGTTTAA	This study
Vp2516D-C1	GTAAGCAACAACAGTTGAAAGAGCTCGTGAG	This study
Vp2516D-D1	GTCTAGAAATCGACGAGCG	This study
VP2516-FLAG-A	CATGCATGCGCTCTGTAATTTGTTTGCC	This study
VP2516-FLAG-B	**TTTATCGTCGTCATCTTTGTAATC**(FLAG) CATATCCATTTTCCTTGCC	This study
VP2516-FLAG-C	**TTTATCGTCGTCATCTTTGTAATC**(FLAG) CATATCCATTTTCCTTGCC	This study
VP2516-FLAG-D	GCTCTAGATTCGTGTTCAAATCTGAGC	This study
VP2516-FLAG-F	GATTACAAAGATGACGACGAT AAA	This study
VP2516-FLAG-R	GATGCGCTCCACTCGAACC	This study
Vp2516D_ICF	GCAAACGATCACCTAATCT	This study
Vp2516D_ICR	GGGTCTAGCTGCTGAAGTA	This study
ChIP-qPCR		
VPAr01_132951_F	GCGGCAGGCCTAACACAT	This study
VPAr01_133200_R	CGCCTTGGTGAGCCCTTAC	This study
VP2762-ChIP-q-F	ATAATTGCGCAGCAAATAACC	This study
VP2762-ChIP-q-R	GCATGAAGTGGAATACTAGGACAA	This study
VPA0606-ChIP-q-F	CTGTAAGTGAAGAATAATACCGAATG	This study
VPA0606-ChIP-q-R	AAGTGGTTAAAGCGAATGATC	This study
qRT-PCR		
VPAr01_443_F	ACTTTCAGTCGTGAGGAAGG	This study
VPAr01_701_R	CACCGCTACACCTGAAATTC	This study
VP2516_279_F	CGCAAACATCACCAATGCGA	This study
VP2516_531_R	TGCTTGTACGAACAGCGAGT	This study
VPA1649 _323_F	CGAATGTACGAACGCTGATG	This study
VPA1649_563_R	AAAGAGTGCTGTAGCCTCAG	This study
VPA0755 _1106_F	CTGGCGGTATTAATGAAGCG	This study
VPA0755_1373_R	GTATTTTCAGTGCCCCAACC	This study
VP0907_815_F	GGCCACACAAATTCTCTCAC	This study
VP0907 _1074_R	CATGTTAACGGGCGTATCAC	This study
VPA0449_421_F	AATGAAGGTCTCGGTGGAAG	This study
VPA0449_681_R	AATGCTACTCACGCTATCCC	This study
VP0432_299_F	TAGCTCAAGCAGACCAAGTC	This study
VP0432_535_R	GACCTGTCGCGGAAATAATG	This study
VPA1467_897_F	GATACTGCCATTGGAACACG	This study
VPA1467_1130_R	GGAACAGAAACACCATCGTG	This study
VPA2032_786_F	CAAAGACTTCATCCGTGAGC	This study
VPA2032_1016_R	CTGTCAGCGACTTTACTTGC	This study
LuxAB assay		
VP0907-luxAB-F1	TGCAGGTTTGACGGCAGAGC	This study
VP0907-luxAB-R1	TCAGGGGTGTCTGAATTACGCT	This study
VPA1649-luxAB-F1	CGGTTCGTAAATGCGTTTTGC	This study
VPA1649-luxAB-R1	CCTCTCGCTCTGAAAGGTCGTT	This study
VPA0755-luxAB-F1	GAAGGTCGCGCGGTCAAAA	This study

VPA0755-luxAB-R1	TGGCTCTAATCGCTCCAACTGA	This study
VP0432-luxAB-F1	CGGAGCTTCGCTCTATTGTTGA	This study
VP0432-luxAB-R1	CAGAACCTAGCCCTTGTGTCGA	This study
VPA0449-luxAB-F1	ATGCGTTTTTTGAGCGGTTGG	This study
VPA0449-luxAB-R1	TCTGCTCTGACGCATCAACTCC	This study
VPA2032-luxAB-F1	TGATGATCTTGTTGTTGCTGCG	This study
VPA2032-luxAB-R1	CCAACTTTTTCGCCAGCAACAT	This study
VPA1467-luxAB-F1	ACAAAAACGTATCGGTGGGTGA	This study
VPA1467-luxAB-R1	TGCGCCAGCAGATCTTGTGT	This study
PSA19CP-F	TGCGTTCGTGTCTTTGAATC	This study
His-OpaR expression		
VP2516-EXF	GCG<u>GGATCC</u>ATGGACTCAATTGCAAAG	This study
VP2516-EXR	GCG<u>AAGCTT</u>TTAGTGTTCGCGATTGTAG	This study
Footprinting assay		
VP0907-footprint-F-6FAM	TGCAGGTTTGACGGCAGAGC	This study
VP0907- footprint-R	TCAGGGGTGTCTGAATTACGCT	This study
VP0907-footprint-F	TGCAGGTTTGACGGCAGAGC	This study
VP0907- footprint-R-6FAM	TCAGGGGTGTCTGAATTACGCT	This study
VPA1649-footprint-F-6FAM	CGGTTCGTAAATGCGTTTTGC	This study
VPA1649- footprint-R	CCTCTCGCTCTGAAAGGTCGTT	This study
VPA1649-footprint-F	CGGTTCGTAAATGCGTTTTGC	This study
VPA1649- footprint-R-6FAM	CCTCTCGCTCTGAAAGGTCGTT	This study
VPA0755- footprint-F-6FAM	GAAGGTCGCGCGGTCAAAA	This study
VPA0755- footprint-R	TGGCTCTAATCGCTCCAACTGA	This study
VPA0755- footprint-F	GAAGGTCGCGCGGTCAAAA	This study
VPA0755- footprint-R-6FAM	TGGCTCTAATCGCTCCAACTGA	This study
VPA0449- footprint-F-6FAM	ATGCGTTTTTTGAGCGGTTGG	This study
VPA0449- footprint-R	TCTGCTCTGACGCATCAACTCC	This study
VPA0449- footprint-F	ATGCGTTTTTTGAGCGGTTGG	This study
VPA0449- footprint-R-6FAM	TCTGCTCTGACGCATCAACTCC	This study
VP0432- footprint-F-6FAM	CGGAGCTTCGCTCTATTGTTGA	This study
VP0432- footprint-R	CAGAACCTAGCCCTTGTGTCGA	This study
VP0432- footprint-F	CGGAGCTTCGCTCTATTGTTGA	This study
VP0432- footprint-R-6FAM	CAGAACCTAGCCCTTGTGTCGA	This study
VPA2032- footprint-F-6FAM	TGATGATCTTGTTGTTGCTGCG	This study
VPA2032- footprint-R	CCAACTTTTTCGCCAGCAACAT	This study
VPA2032- footprint-F	TGATGATCTTGTTGTTGCTGCG	This study
VPA2032- footprint-R-6FAM	CCAACTTTTTCGCCAGCAACAT	This study
VPA1467- footprint-F-6FAM	ACAAAAACGTATCGGTGGGTGA	This study
VPA1467- footprint-R	TGCGCCAGCAGATCTTGTGT	This study
VPA1467- footprint-F	ACAAAAACGTATCGGTGGGTGA	This study
VPA1467- footprint-R-6FAM	TGCGCCAGCAGATCTTGTGT	This study
EMSA		
VP0907-EMSA-F1	AGTACATCATGATCGTCGTG	This study
VP0907-EMSA-R1	GCATTCATTTTGCTTGTTAC	This study
VP0907-EMSA-F2	GAATTTAAAGTGATATTTTG	This study
VP0907-EMSA-R2	TACAGAAATGAAAGGATAAG	This study
VPA1649-EMSA-F1	ACTGGCTAATGAGTGCTCTA	This study

VPA1649-EMSA-R1	GGTTTATCCTATAAATTGCG	This study
VPA1649-EMSA-F2	CGTCACTCTAAAGTGCCAAAC	This study
VPA1649-EMSA-R2	GGCAAATTGGAAAGAAGTGTC	This study
VPA0755-EMSA-F1	CAGTCAGTAAAGCGGCACC	This study
VPA0755-EMSA-R1	GTTTGTGTGACTCATATCGCTTAC	This study
VPA0449-EMSA-F1	GTTAGCTCCCGCCCATTTCCT	This study
VPA0449-EMSA-R1	TACGGCTCAGCCCCCATCAA	This study
VP0432-EMSA-F1	CGATTGATTCATCACTCTTGGTG	This study
VP0432-EMSA-R1	GCGCCAAAATGGCATAGAAAG	This study
VPA2032-EMSA-F1	CAGTGTTAGCCGTTCATTACGT	This study
VPA2032-EMSA-R1	CTAATCTGTTAACCAAAAACTGGTG	This study
VPA1467-EMSA-F1	CACTTAGCCAACCCTTGTCTTT	This study
VPA1467-EMSA-R1	AACCAGTGTAAACACAATCAACATC	This study
VPA1467-EMSA-F2	CGTTTTTTCAAGCAGGTTAGA	This study
VPA1467-EMSA-R2	CATTTATAGGATTAATGGAATCCG	This study

[a]Underline: sequence of restriction enzyme sites, Bold: FLAG-tag sequence

Figure S1

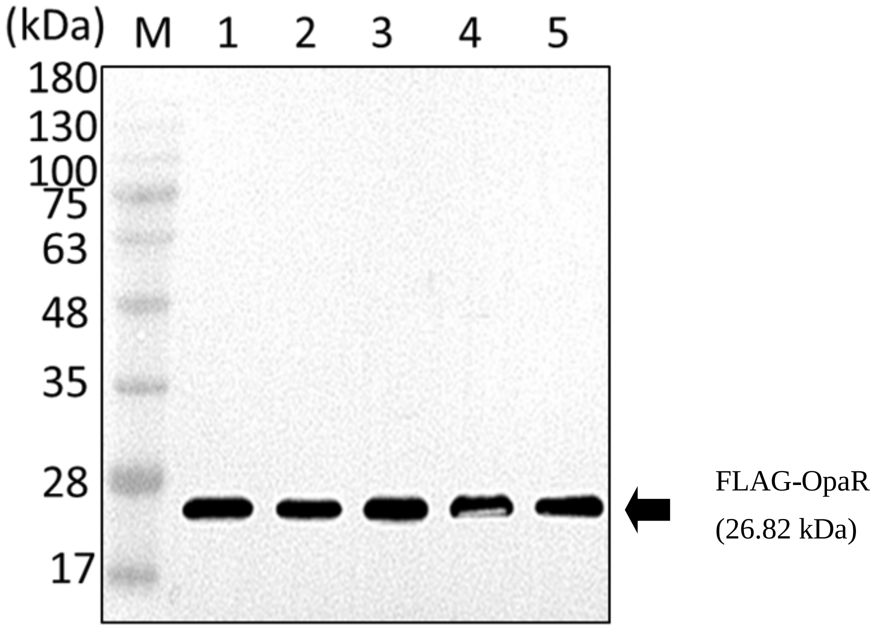

Figure S1 Expression levels of FLAG-OpaR at different growth phases.
Whole cell extract of FLAG-OpaR/VP93 was collected. Expression level of FLAG-OpaR was detected using Western blotting with anti-FLAG antibody in the early-log (lane 1: OD_{600} 0.2), log (lane 2: OD_{600} 0.5), late-log (lane 3: OD_{600} 2.8), stationary (lane 4: OD_{600} 4), and late-stationary (lane 5: OD_{600} 5.2) phases. Marker, Prestained Protein Ladder 1025G (Bioman).
